# Activity-dependent plasticity of mouse hippocampal assemblies *in vitro*

**DOI:** 10.3389/fncir.2015.00021

**Published:** 2015-05-18

**Authors:** Martin K. Keller, Andreas Draguhn, Martin Both, Susanne Reichinnek

**Affiliations:** ^1^Institute of Physiology and Pathophysiology, University of HeidelbergHeidelberg, Germany; ^2^Mediterranean Institute of Neurobiology (INMED), INSERMMarseille, France

**Keywords:** plasticity, sharp wave-ripple, SPW-R, oscillation, hippocampus, assembly, neuronal assemblies, neuronal plasticity

## Abstract

Memory formation is associated with the generation of transiently stable neuronal assemblies. In hippocampal networks, such groups of functionally coupled neurons express highly ordered spatiotemporal activity patterns which are coordinated by local network oscillations. One of these patterns, sharp wave-ripple complexes (SPW-R), repetitively activates previously established groups of memory-encoding neurons, thereby supporting memory consolidation. This function implies that repetition of specific SPW-R induces plastic changes which render the underlying neuronal assemblies more stable. We modeled this repetitive activation in an *in vitro* model of SPW-R in mouse hippocampal slices. Weak electrical stimulation upstream of the CA3-CA1 networks reliably induced SPW-R of stereotypic waveform, thus representing re-activation of similar neuronal activity patterns. Frequent repetition of these patterns (100 times) reduced the variance of both, evoked and spontaneous SPW-R waveforms, indicating stabilization of pre-existing assemblies. These effects were most pronounced in the CA1 subfield and depended on the timing of stimulation relative to spontaneous SPW-R. Additionally, plasticity of SPW-R was blocked by application of a NMDA receptor antagonist, suggesting a role for associative synaptic plasticity in this process. Thus, repetitive activation of specific patterns of SPW-R causes stabilization of memory-related networks.

## Introduction

The mammalian hippocampus supports the formation and consolidation of spatial, episodic and declarative memories. At the network level, neuronal activity is organized in coherent spatio-temporal patterns which correlate with distinct processes in memory formation (Sirota and Buzsáki, 2005). Recording of multiple single units in freely moving rodents has revealed that hippocampal network oscillations go along with precisely timed action potentials of specific neurons which fire in a place-specific manner. Sequential activation of such place cells during spatial exploration forms a representation of the animal’s trajectory (O’Keefe and Recce, 1993). In subsequent phases of slow-wave sleep, the same sequences are repetitively activated, probably mediating memory consolidation (Wilson and McNaughton, 1994; Lee and Wilson, 2002). In this state, sequential activation of place cells occurs on top of sharp wave-ripple complexes (SPW-R; Ylinen et al., 1995; Csicsvari et al., 1999). It has been suggested that this re-activation of neuronal activity sequences mediates memory consolidation by transferring transiently stable activity into the neocortex for long-term storage (Buzsáki, 1989). In line with this concept, memory consolidation is associated with irregular oscillatory activity containing SPW-R (Girardeau et al., 2009; Mölle and Born, 2011; Sadowski et al., 2011).

Work during the past decades has revealed much insight into the cellular mechanisms underlying memory formation, including Hebbian plasticity of synapses in major hippocampal projection pathways (Bliss and Lomo, 1973; Köhr et al., 2003; Buschler et al., 2012; Goh and Manahan-Vaughan, 2013). Oscillation-associated assembly formation constitutes a potential link between synaptic plasticity and network-level information processing. Indeed, theta-gamma states support synaptic plasticity (Palmer et al., 2004; Lisman et al., 2005) and may thus allow for transient assembly formation. In accordance, *in vitro* data show that high frequency stimulation can induce spontaneous SPW-R oscillations in rat hippocampal slices (Behrens et al., 2005). Additionally, this induction is NMDA-R activation dependent and indicates an essential role of synaptic strengthening in hippocampal assembly formation. Interestingly, SPW-Rs also favor associative strengthening of synapses (King et al., 1999), and their selective disruption impairs spatial memory formation (Girardeau et al., 2009). Together, these processes may account for the rapid formation and subsequent consolidation of space-encoding assemblies in hippocampal networks (Frank et al., 2003; Carr et al., 2012). It is, thus, likely that repeated activation of assemblies during SPW-R strengthens connectivity between participating neurons, resulting in transiently stable, highly specific activity patterns (Buzsáki and Draguhn, 2004). While we know much about plasticity of synapses, the activity-dependent formation of neuronal assemblies within the different hippocampal subfields remains, however, poorly understood.

In previous work we showed that different neuronal assemblies are reflected in different waveforms of spontaneously occurring SPW-R (Reichinnek et al., 2010). Therefore, extracellular recordings of SPW-R (Maier et al., 2003) and subsequent waveform sorting can be taken as a proxy of monitoring distinct neuronal assemblies. Here, we tested the effect of repetitive activation of such neuronal groups on the diversity and stability of spontaneous network activity patterns.

## Materials and Methods

All animal procedures were performed in accordance with the guidelines of the European Community Council and approved by the state government of Baden-Württemberg.

### Experimental Outline

#### Slice Preparation

Experiments were performed on slices of four to 8 weeks old male mice (C57Bl6). Animals were decapitated after being deeply anesthetized with CO_2_. Subsequently, the brain was separated and transferred into cooled (0–4°C) artificial cerebral spinal fluid (ACSF: NaCl 124 mM, KCl 3.0 mM, MgSO_4_ 1.8 mM, CaCl_2_ 1.6 mM, Glucose 10 mM, NaH_2_PO_4_ 1.25 mM, NaHCO_3_ 26 mM), saturated with 95% O_2_ and 5% CO_2_ (corresponding to pH 7.4 at 37°C). Before slicing, frontal lobe and cerebellum were removed, and the remaining tissue block was glued to the holding chamber of a Leica Vibratome (VT1000S). Horizontal slices of 450 μm thickness were cut and subsequently transferred to a Haas-type interface recording chamber where they recovered for at least 2 h at 32–34°C at the border between ACSF (flow: 1.5–2.0 ml/min) and humidified gas (95% O_2_ and 5% CO_2_).

#### Recording and Stimulation

Extracellular field potentials were recorded in stratum pyramidale of CA3a, CA3b, and CA1 by use of three tetrodes, each consisting out of four twisted wires (Tungsten California Fine Wire, 12.5 μm) connected to an EXT-T2 amplifier (npi electronics, Tamm, Germany). Signals were amplified (×200), low pass filtered at 8 kHz and digitized at 20 kHz for offline analysis (CED Power 1401 mkII expanded by a CED 2805SA-8 Analog BCN box, recording program Spike 2.0, CED, Cambridge, UK).

**Figure 1:**
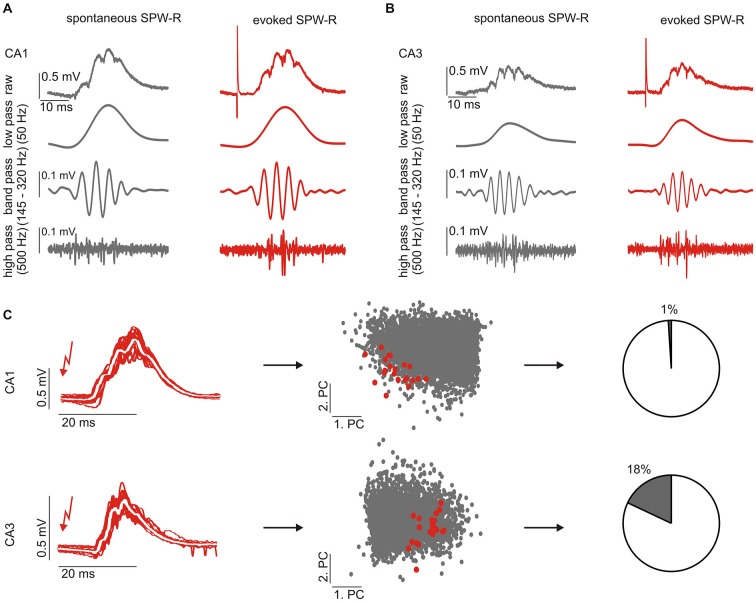
**Electrically evoked sharp wave-ripple complexes (SPW-R) closely resemble spontaneous events. (A,B)** Example traces of spontaneous (left) and evoked (right) SPW-R in CA1 **(A)** and CA3 **(B)**, respectively. Evoked SPW-R show the same features like spontaneous SPW-R in different frequency bands corresponding to the sharp wave (low pass), ripple oscillations (band pass) and multi-unit activity (high pass). **(C)** Individual SPW-R waveforms (left) were analyzed by principal component analysis (PCA) and their similarity was quantified in the first 10 dimensions of the principal component (PC) space (middle, only two PCs are shown). Red dots indicate individual evoked SPW-R shown on the left panel, gray dots indicate spontaneous SPW-R. A smaller inter-group pair-wise average distance indicates more similar waveforms. To compare evoked and spontaneous events we quantified the overlap of events (right, see Methods).

Two stimulation electrodes (“Micro Probes” Platinum/Iridium, 100 kΩ at 1 kHz, 75 μm tip separation) were placed into the granule cell layer of the supra-pyramidal blade of area dentata. Electrode positions and stimulation strengths were optimized such that unipolar stimulation pulses evoked field events in CA3 and CA1 which resembled waveforms of spontaneously occurring SPW-R (Figures 1A,B). In order to avoid direct interference with ongoing network activity, pulses were triggered at a delay of 150 ms after a preceding spontaneous SPW-R waveform. Alternating stimulation of both electrodes (at intervals of 60 s) caused two different waveform sets of evoked SPW-R at recording sites in CA3a, CA3b and CA1. After 20 stimulations performed at each site, we applied a series of 100 repetitive stimulations at 10 s intervals to one of the two stimulation electrodes, followed again by 20 alternating stimulations at both sites. The first group of control slices underwent the baseline stimulation protocol (alternating stimulation at both sites at intervals of 60 s) but not the repetitive stimulation at 0.1 Hz (1000 s pause at the respective time). Since there was no repetitively stimulated and not repetitively stimulated pattern in this condition we subsequently recorded two evoked waveform data sets in each slice. In a second control condition the 100 repetitive stimulations were triggered without delay on a spontaneously occurring SPW-R while baseline stimulations were still performed with a delay of 150 ms. In a third control the original experiment was repeated in the presence of the NMDA-R antagonist APV (30 μM).

### Data Analysis

#### Analysis of Field Potentials

Raw data were analyzed with custom-made routines written in Matlab. To extract SPW-R events, data were low-pass filtered (50 Hz) and local maxima with amplitudes above 0.15 mV were detected within 30 ms time windows. This threshold corresponds to ~4 standard deviations of event-free baseline noise (Both et al., 2008) and allows for reliable detection of SPW-R. Identified SPW-Rs were analyzed within a time interval starting 33 ms before and ending 67 ms after the peak of the sharp wave. Waveforms of evoked SPW-R-like events were processed similarly to spontaneous SPW-R after removal of the stimulus artifact from raw data traces. Extracellular multi-unit activity was detected by high-pass filtering at 500 Hz and setting a negative threshold at 4.5 SDs from background noise.

The stability of the extracellular signal was controlled by monitoring amplitude and frequency of spontaneous SPW-R during baseline recording. These parameters were regarded stable when rejecting the null hypothesis of finding a change at 5% significance level according to the Wald-Wolfowitz test. In some slices stimulation failed to evoke a downstream response pattern at one site during the course of the experiment. We suspected this to be a local effect since stimulation at the other site and spontaneous SPW-Rs were not affected. Subsequently we excluded experiments with less than ten evoked waveforms to ensure sufficient numbers of evoked events.

#### Waveform Analysis

For analysis of SPW-R waveforms, signals were down-sampled to 5 kHz, resulting in 500 sample points within a 100 ms window. This data set was further reduced by principal component analysis (PCA; Nicolelis et al., 1995; Lopes-dos-Santos et al., 2013). In accordance with our previous work (Reichinnek et al., 2010) we used the first 10 principal components to describe events within a 10 dimensional feature space. The first PC mainly reflected waveform amplitude and was divided by two in order to reduce the relative contribution of this parameter.

Similarity or difference of waveforms was analyzed by calculating the pairwise Euclidian distance between waveforms within the 10-dimensional principal component space. Subsequently, we calculated the pair-wise Euclidian distance between all spontaneous as well as evoked events before and after repetitive stimulation. This resulted in distance distributions for evoked and spontaneous events, respectively. To compare changes in the variety of waveforms, we divided the median distance after the repetitive stimulation by the median distance before the repetitive stimulation.

In a different approach, we measured the overlap between the repetitive evoked waveform distribution and the evoked SPW-Rs from the site without repetitive stimulation. To quantify this overlap we calculated the distances between the group of evoked but not repetitively stimulated events and the distribution of the group of evoked and repetitively stimulated events. In this case, we used the Mahalanobis distance which is a multi-dimensional way of measuring how many standard deviations a specific event is distant from the center of a distribution. Events from the group of evoked but not repetitively stimulated events were considered similar to the repetitive stimulated SPW-R waveform distribution if the Mahalanobis distance was smaller than the first percentile of the Mahalanobis distances within the distribution of the group of repetitively stimulated events itself. Subsequently, the overlap between both groups of evoked events was defined as the percentage of events from the group of evoked events that were not repetitively stimulated but were within the distribution of evoked events that underwent repetitive stimulation. In analogy the overlap between spontaneous SPW-R and the repetitive stimulated waveform pattern was calculated.

#### Statistics

Quantitative results are given as median, the 25^th^ and the 75^th^ percentiles. Whiskers in the box plots correspond to the 2.5^th^ and 97.5^th^ percentiles. For statistical analysis we tested whether data were normally distributed by Kolmogorov-Smirnoff-Test (significance level > 0.05). Paired normally distributed data were analyzed by paired *t*-test, whereas non-paired data were tested by two sided *t*-test. Non-normally distributed data were compared by Wilcoxon signed-rank test and Wilcoxon rank sum test, respectively. In all cases, *p* < 0.05 was regarded as significant.

## Results

### Weak Stimulation of the Dentate Gyrus Elicits Physiological SPW-R Waveforms

Compound potentials similar to spontaneous SPW-R can be evoked by weak electrical stimulation in CA3 (Reichinnek et al., 2010). In order to evoke such events in both hippocampal subfields, CA3 and CA1, we stimulated the upstream network of the DG which is powerfully connected to CA3 pyramidal cells and inhibitory neurons (Acsády et al., 1998). To avoid interference with the ongoing network activity stimuli were applied at 150 ms after the occurrence of a spontaneous SPW-R event. Under these conditions, weak electrical stimulation within the supra-pyramidal blade of the granule cell layer (100 μs, 0.5–5 V) reliably evoked field potential transients in the pyramidal cell layers of CA3 (*n* = 12 slices) and CA1 (*n* = 10 slices), respectively. These transient potentials resembled spontaneous sharp wave-ripple events and had characteristic, rather stereotypic waveforms (Figures 1A,B). Although we observed a lower number of unit-events on evoked waveforms overall quantitative analysis further confirmed the similarity between evoked field events and spontaneous SPW-R (Table 1). Moreover, it should be noted that spontaneously occurring SPW-R show heterogeneous levels of multi-unit activity not reflected by the median value across slices.

**Table 1:** **Spontanoues (top) and evoked (bottom) waveforms are compared regarding amplitude (left), ripple occurrence (middle), and unit occurrence (right) in CA1 and CA3**.

	SPW-R amplitude (mV)		Number of ripple cycles (per SPW-R)		Multi-unit activity (spikes per SPW)	
	CA1	CA3	CA1	CA3	CA1	CA3
Spontaneous events	median 0.23	median 0.21	median 6.0	median 5.5	median 3.0	median 4.4
	25^th^ 0.17	25^th^ 0.17	25^th^ 5.7	25^th^ 3.6	25^th^ 1.9	25^th^ 1.2
	75^th^ 0.28	75^th^ 0.25	75^th^ 6.2	75^th^ 6.5	75^th^ 4.3	75^th^ 10
Evoked events	median 0.26	median 0.29	median 5.8	median 4.7	median 1.0	median 0.8
	25^th^ 0.19	25^th^ 0.19	25^th^ 5.6	25^th^ 4.0	25^th^ 0.95	25^th^ 0.63
	75^th^ 0.61	75^th^ 0.37	75^th^ 6.9	75^th^ 5.4	75^th^ 1.0	75^th^ 1.0
*p*-value	0.14	0.0051	0.38	0.10	0.096	0.10
	(*n* = 10)	(*n* = 12)	(*n* = 10)	(*n* = 12)	(*n* = 10)	(*n* = 12)

*Median, 25^th^ percentile, and 75^th^ percentile represent the distribution formed by the medians observed in each experiment and p-values (bottom line) are calculated between spontanoues and evoked events*.

For further comparison of spontaneous and evoked waveform patterns we described the events by a reduced set of parameters following principal component analysis (PCA, see Methods). The principal components describing evoked events in both hippocampal subfields (CA1 and CA3) were reliably located within the parameter space covered by spontaneous events (Figure 1C), which underlines the similarity of both patterns. In summary, we conclude that stimulation-evoked network events in CA3 and CA1 are very similar to spontaneous ongoing spontaneous SPW-R activity and are therefore referred to as “evoked SPW-R”.

### Evoked SPW-R Waveforms are Stabilized by Repetitive Stimulation

**Figure 2:**
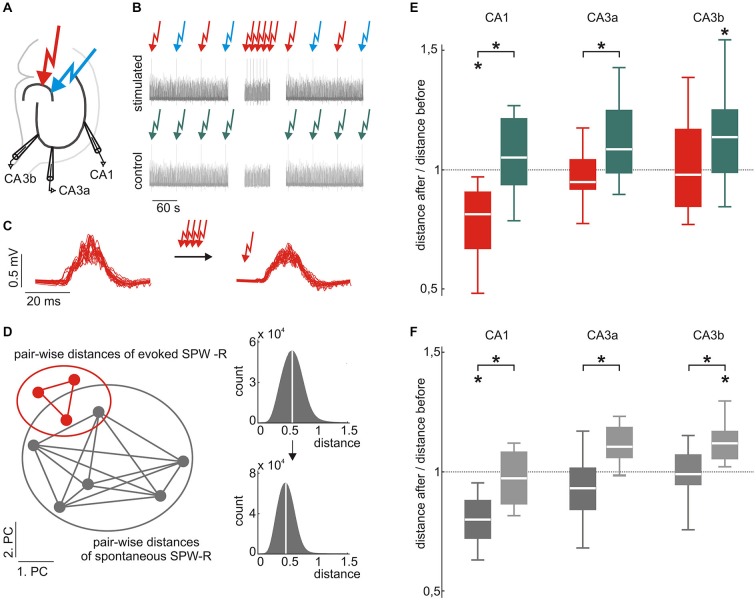
**Evoked and spontaneous SPW-R waveforms are stabilized by low frequency stimulation. (A)** Scheme of the electrode positions. Recording electrodes were located in CA3a, CA3b, and CA1. Two different locations in the dentate gyrus (DG) were electrically stimulated to evoke SPW-R complexes. **(B)** Scheme of the stimulation paradigm (top) and the control (bottom). Red and blue arrows indicate electrical stimulation at two different locations in the DG. Low-frequency repetitive stimulation (100 stimuli every 10 s, middle part) was only performed at one of the locations. Under control conditions, stimulation was paused instead for 1000 s. **(C)** Example of evoked SPW-R before (left) and after (right) repetitive stimulation. **(D)** Homogeneity of evoked and spontaneous SPW-R was quantified by the median intra-group pair-wise Euclidian distance in the 10-dimensional PC space. Smaller median distances indicate more similar waveforms. **(E)** Evoked SPW-R waveforms in CA1 became more similar after repetitive stimulation (red; median 0.81, 25^th^ 0.66, 75^th^ 0.90, *p* < 0.05, *n* = 8), whereas events under control conditions did not (green; median 1.05, 25^th^ 0.93, 75^th^ 1.21, *n* = 11 data sets from 7 slices), which resulted in a significant group difference (*p* < 0.01). Similarly, in CA3a we found a significant difference between both conditions (*p* < 0.05, repetitive stimulation: median 0.95, 25^th^ 0.91, 75^th^ 1.04, *n* = 8; control: median 1.08, 25^th^ 0.98, 75^th^ 1.25, *n* = 11 data sets from 6 slices). In CA3b repetitive stimulation (median 0.97, 25^th^ 0.84, 75^th^ 1.17, *n* = 12) prevented an increase of variability that was observed under control conditions (median 1.14, 25^th^ 0.98, 75^th^ 1.25, *p* < 0.05, *n* = 17 data sets from 9 slices). **(F)** Spontaneous events also showed a reduced variability after repetitive stimulation of evoked events as compared to control conditions (*p* < 0.05 for all subfields). Additionally, spontaneous events in CA1 became significantly less variable after repetitive stimulation (repetitive stimulation: median 0.80, 25^th^ 0.72, 75^th^ 0.88, *p* < 0.01, *n* = 10; control: median 0.97, 25^th^ 0.86, 75^th^ 1.08). Moreover, spontaneous events in CA3 became more diverse under control conditions (CA3a: median 1.10, 25^th^ 1.06, 75^th^ 1.18, *p* = 0.075, *n* = 6; CA3b: median 1.12, 25^th^ 1.06, 75^th^ 1.17, *p* < 0.01, *n* = 9), which was inhibited by 100 repetitive stimulations (CA3a: median 0.93, 25^th^ 0.84, 75^th^ 1.02; CA3b: median 0.99, 25^th^ 0.95, 75^th^ 1.07). “*” means that the finding is significant (*p* < 0.05).

Memory consolidation has been suggested to depend on the repetitive re-play of hippocampal activity patterns during SPW-R (Wilson and McNaughton, 1994; Lee and Wilson, 2002; Girardeau et al., 2009). We therefore asked for the effect of repetitive activation of similar sets of neurons through electrical stimulation. Twenty highly stereotypic SPW-R waveforms were evoked every 60 s alternating at two stimulation sites within the DG. Subsequently, one site was selected and 100 repetitive pulses were performed at 10 s intervals followed by 20 more stimulations every 60 s at both sites (Figure 2A). Stability of evoked waveform sets prior and after the 100 repetitive stimulation pulses was analyzed within the 10-dimensional PCA parameter space by calculating the pair-wise Euclidian distance between individual SPW-R.

In CA1, median pair-wise distances of evoked SPW-R were consistently reduced following the 100 repetitive stimulations. This was quantified by the ratio of median distances after/before stimulation which was 0.81 (*p* = 0.029, *n* = 8; Figure 2D). In contrast, slices that did not undergo the 100 repetitive stimulations (1000 s stimulation pause respectively) showed no reduction in waveform variability (ratio of median distances late/early recording period 1.05, *p* = 0.25, *n* = 11 data sets from 7 slices). The difference between both conditions was significant (*p* = 0.0023) indicating that repetitive activation mediates stabilization of evoked SPW-R waveform patterns in CA1. Within CA3, SPW-Rs were recorded at two different sites (CA3a and CA3b). Under control conditions (1000 s stimulation pause), waveforms in CA3a showed a trend towards increased diversity over time (median distances late/early recording period 1.08, *p* = 0.051, *n* = 11 data sets from 6 slices), which reached significance in CA3b (median 1.14, *p* = 0.016, *n* = 17 data sets from 9 slices). The increase in variance in both CA3 regions was prevented by 100 repetitive stimulations within the DG, which is similar to the results obtained in CA1. In stimulated slices, median distance after/before repetitive stimulations was 0.95 (*p* = 0.72, *n* = 8) in CA3a and in CA3b, respective value was 0.97 (*p* = 0.55, *n* = 12). When compared to un-stimulated slices, the reduction of variability was significant for CA3a (*p* = 0.045) while the ratio of waveform distances in CA3b did not reach significance (*p* = 0.10).

As stated above, baseline stimulation was alternating between two different sites within the supra-pyramidal blade of the DG. Repetitive stimulation at 0.1 Hz (100 pulses) was, however, restricted to one of the two locations. Interestingly, SPW-R waveforms in CA1 elicited from the other site (which was not repetitively stimulated) did also show reduced variance following the 100 repetitive stimulations at the other site. This was reflected by the ratio of the median distances after/before stimulation of 0.66 (25^th^ 0.64, 75^th^ 0.75, *p* = 0.0039, *n* = 7, *p* = 0.00037 in comparison to the “un-stimulated” control condition). In CA3, similar to the site which was repetitively stimulated, evoked events at the site which was not repetitively stimulated did not show any change in variability (CA3a ratio after/before median 1.01, 25^th^ 0.92, 75^th^ 1.31, *p* = 0.67, *n* = 8, *p* = 0.71 in comparison to “un-stimulated” control condition; CA3b, ratio after/before median 1.01, 25^th^ 0.84, 75^th^ 1.09, *p* = 0.76, *n* = 8, *p* = 0.051 in comparison to the “un-stimulated” control condition).

### Spontaneous Events Mimic Evoked Events and Reduce Their Variability

Surprisingly, reduction of waveform variability after 100 repetitive stimulations was also observed for spontaneous SPW-R in CA1 and CA3. Median Euclidian distances of spontaneously occurring events in CA1 were significantly smaller in slices that had undergone 100 repetitive stimulation pulses as compared to “un-stimulated” slices (*p* = 0.0044). Accordingly, the ratio of distances after/before stimulation was 0.80 (*p* = 0.0035, *n* = 10) in the repetitively stimulated slices. In “un-stimulated” control condition this value was 0.97 (*p* = 0.64, *n* = 7). In CA3, variability of spontaneous SPW-R increased under control conditions, similar to the evoked events (CA3a: median 1.10, *p* = 0.075, *n* = 6; CA3b: median 1.12, *p* = 0.0054, *n* = 9). Again, this progressive increase in variability was reduced by 100 intermittent repetitive stimulations (CA3a: median 0.93, *p* = 0.24, *n* = 10; CA3b: median 0.99, *p* = 0.82, *n* = 12). The differences in variability ratios between the repetitively stimulated condition and the “un-stimulated” control were significant in CA3a (*p* = 0.015) and CA3b (*p* = 0.0069), respectively.

Overall, these results indicate that repetitive activation of limited neuronal subsets in the hippocampal formation reduces variance of subsequent network patterns, indicating activity-dependent stabilization of hippocampal assemblies.

### Stimulation-Evoked Waveform Sets Increase Similarity

**Figure 3:**
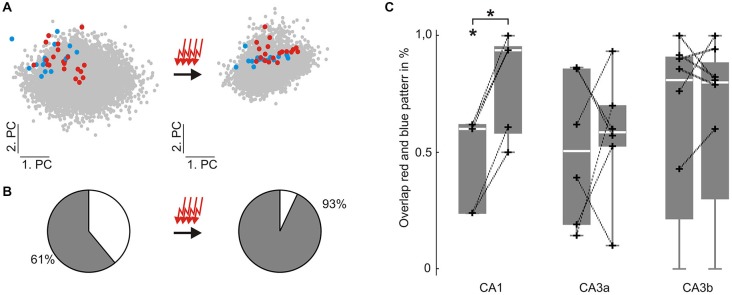
**Similarity between two different groups of evoked SPW-R increases in CA1 but not in CA3 following repetitive stimulation. (A)** colored dots indicate individual evoked SPW-R waveforms depicted in two dimensions of the principal component (PC) space before (left) and after (right) 100 repetitive stimulations. Red and blue dots are evoked by electrical stimulation at two different locations within the DG. Only the location corresponding to the red dots is repetitively stimulated. Gray dots indicate spontaneous SPW-R. **(B)** Similarity between the two different groups of evoked events was quantified by their overlap in the 10-dimensional PC space. **(C)** Overlap of the two groups of evoked events was significantly increased in CA1 (initial overlap: median 60%, 25^th^ 24%, 75^th^ 62%; past stimulation: median 94%, 25^th^ 58%, 75^th^ 95%, *p* < 0.0001, *n* = 5). However, in CA3 overlap between both stimulation-induced patterns did not change (CA3a: initial overlap: median 51% 25^th^ 19%, 75^th^ 86%; past stimulation: median 59%, 25^th^ 53%, 75^th^ 70%; CA3b: initial overlap: median 81% 25^th^ 21%, 75^th^ 91%; past stimulation: median 80%, 25^th^ 30%, 75^th^ 88%). “*” means that the finding is significant (*p* < 0.05). “+” represent the original data underlying the distribution.

Since evoked waveform sets from both stimulation sites became more homogeneous despite the fact that only one location was repetitively stimulated 100 times (see Figure 2), we analyzed the distinctiveness of both patterns. To do this, we calculated the Mahalanobis distance between both groups of evoked events within the principal component space (see Methods). In CA1 we found an initial overlap of 60%, which significantly increased to 94% (*p* = 0.000095, *n* = 5) by the 100 repetitive stimulations (see Figure 3). In contrast, in CA3 the overlap between both evoked patterns did not change significantly. In CA3a the initial overlap was 51% before, and 59% after the 100 stimulations (*p* = 0.71, *n* = 6). In CA3b we observed an overlap of 81% prior to and 80% after the repetitive stimulation (*p* = 0.80, *n* = 8, Figure 3C).

In slices that had not undergone 100 repetitive stimulations, the overlap between events was calculated in both directions (data not shown). In contrast to the stimulated condition, overlap remained stable in CA1 (*p* = 0.25, *n* = 10 data sets within 5 slices) and percentages of overlap were lower than in the above described repetitive stimulation paradigm (initial overlap: median 17%, 25^th^ 5%, 75^th^ 48%; after stimulation pause: median 5%, 25^th^ 5%, 75^th^ 32%). Likewise, CA3 overlap did not change significantly (CA3a: *p* = 0.19, *n* = 10 data sets within 5 slices; CA3b: *p* = 0.38, *n* = 16 data sets within 8 slices; CA3a initial overlap 38%, 25^th^ 31%, 75^th^ 88%; past stimulation pause 28%, 25^th^ 13%, 75^th^ 60%; CA3b initial overlap 33%, 25^th^ 6%, 75^th^ 78%; past stimulation pause 22%, 25^th^ 2%, 75^th^ 50%).

In summary, both evoked SPW-R waveform sets in CA1 became more similar to each other when one of the stimulation locations was repetitively stimulated 100 times. No significant change in similarity could be observed in CA3 for the repetitive stimulation paradigm. Moreover, similarity of SPW-R waveforms remained the same in the whole hippocampus, when no repetitive stimulation was performed.

### SPW-R Stabilization is Affected by the Timing of Stimulation

**Figure 4:**
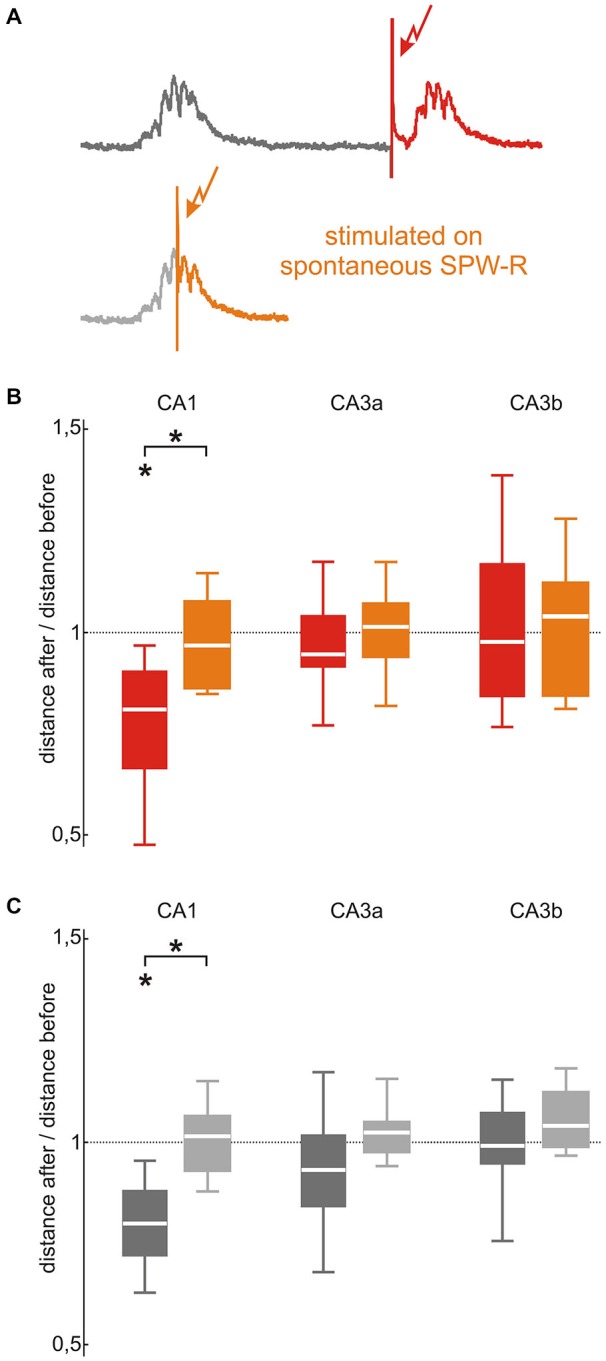
**SPW-R stabilization depends on the timing of the stimulation in CA1 but not in CA3. (A)** In the original stimulation paradigm (upper trace, red) stimulations were triggered 150 ms after spontaneous SPW-R. To investigate the effect of stimulation timing, repetitive stimulations were performed during spontaneous SPW-R in a second control paradigm (lower trace, orange). **(B)** The stabilizing effect of the repetitive stimulation performed 150 ms after a spontaneous event (red; *p* < 0.05, *n* = 8) abolished by stimulating during a spontaneous SPW-R in CA1 (orange; median 0.96, 25^th^ 0.85, 75^th^ 1.07, *n* = 7, *p* < 0.05 between both conditions). This difference was absent in the CA3 (CA3a: median 1.01, 25^th^ 0.93, 75^th^ 1.07; CA3b: median 1.03, 25^th^ 0.84, 75^th^ 1.12) region. **(C)** Spontaneously occurring SPW-R showed the same effect as evoked events: In CA1 the stabilizing effect of repetitive stimulation 150 ms after spontaneous events (dark gray; *p* < 0.01, *n* = 10) was abolished by stimulating during spontaneous SPW-R (light gray; median 1.01, 25^th^ 0.93, 75^th^ 1.07, *n* = 8, *p* < 0.001 between both conditions) but not in CA3 (CA3a: median 1.02, 25^th^ 0.97, 75^th^ 1.05; CA3b: median 1.04, 25^th^ 0.99, 75^th^ 1.12). “*” means that the finding is significant (*p* < 0.05).

Electrical stimulation during an ongoing SPW-R did not elicit a typical SPW-R waveform. This situation can be used to distinguish between the effects of the electrical stimulus itself and the effects of pattern repetition. We therefore tested the effects of 100 repetitive stimulations (~0.1 Hz) timed on a spontaneously occurring SPW (Figure 4A) and compared it to the above described stimulation paradigm (SPW-R evoked by stimulating 150 ms after a spontaneous event). In CA1, stimulating during spontaneous SPW-R did not induce any change in evoked or spontaneous SPW-R waveforms (evoked: median 0.96, *p* = 0.75, *n* = 7; spontaneous: median 1.01, *p* = 0.99, *n* = 8). Subsequently, when compared to the above described original repetitive stimulation paradigm (100 times, 150 ms after spontaneous SPW-R at 0.1 Hz) we found significant difference for evoked (*p* = 0.024) and spontaneous (*p* = 0.00035) waveform stability (Figures 4B,C).

In CA3, 100 repetitive stimulations during an ongoing spontaneous SPW-R did not induce changes in SPW-R waveforms of spontaneous or evoked events (Figures 4B,C). Thus, findings were similar to the ones from the above-described 100 repetitive stimulation 150 ms after a spontaneous SPW-R (CA3a *p* = 0.66 for evoked and *p* = 0.12 for spontaneous events; CA3b *p* = 0.90 for evoked events and *p* = 0.19 for spontaneous events). In CA3a, the after/before distance ratios of evoked (median 1.01, *p* = 0.80, *n* = 6) and spontaneous SPW-R (median 1.02, *p* = 0.30, *n* = 8) were stable. Neither in CA3b SPW-R variability alterations were observed for evoked (median 1.03, *p* = 0.79, *n* = 7) nor spontaneous SPW-R (median 1.04, *p* = 0.11, *n* = 8).

### Modulation of SPW-R Waveform Stability Depends on NMDA Receptor Activation

**Figure 5:**
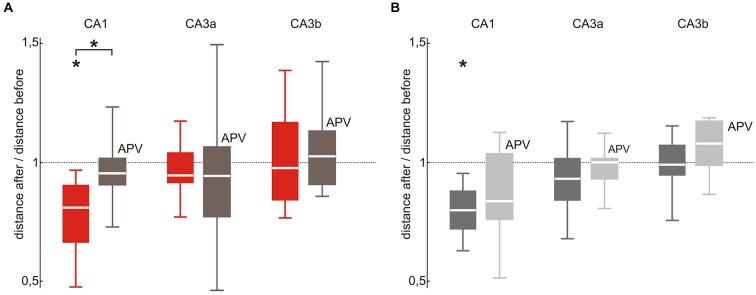
**SPW-R stabilization depends on NMDA receptor activation in CA1 but not in CA3. (A)** In CA1 the stabilizing effect of repetitive stimulation (red; *p* < 0.05 *n* = 8) was abolished by bath application of the NMDA receptor blocker APV (brown; median 0.96, 25^th^ 0.91, 75^th^ 1.0, *n* = 6, *p* < 0.05 between both conditions). This difference was absent in the CA3 (CA3a: median 0.95, 25^th^ 0.78, 75^th^ 1.07; CA3b: median 1.03 25^th^ 0.91, 75^th^ 1.14) region. **(B)** The variability change of spontaneous SPW-R observed after 100 repetitive stimulations in CA1 (dark gray; *p* < 0.01 *n* = 10) did not occur under bath application of APV (light gray; CA1: median 0.84, 25^th^ 0.77, 75^th^ 1.05, *p* = 0.51, *n* = 8). However, there was no significant different across paradigms. Neither in CA3 repetitive stimulation under bath application of APV altered spontaneous SPW-R variability (CA3a: median 1.0, 25^th^ 0.94, 75th 1.02; CA3b: median 1.08, 25^th^ 0.99, 75^th^ 1.18). “*” means that the finding is significant (*p* < 0.05).

Finally, we asked whether NMDA receptor-dependent synaptic plasticity plays a role for stimulation-induced changes of hippocampal assemblies. We therefore repeated the original repetitive stimulation paradigm under bath application of the NMDA receptor antagonist APV (30 μM) over the entire course of the experiment (Figure 5). Under these conditions, median pair-wise distances of evoked events were not affected by 100 repetitive stimulations (CA1 median 0.96, *p* = 0.41, *n* = 6; CA3a median 0.95, *p* = 0.28, *n* = 7; CA3b median 1.03, *p* = 0.54, *n* = 7). Likewise, spontaneously occurring SPW-R remained stable (CA1 median 0.84, *p* = 0.051, *n* = 8; CA3a median 1.0, *p* = 0.56, *n* = 7; CA3b median 1.08, *p* = 0.16, *n* = 8). Compared to the original stimulation paradigm in absence of APV evoked SPW-R stability was significantly lower in CA1 (*p* = 0.047) but not in CA3 (CA3a *p* = 0.89; CA3b *p* = 0.68). Interestingly, we found no significant difference in spontaneous SPW-R variability between slices recorded with to slices recorded without APV bath application (CA1 *p* = 0.33; CA3a *p* = 0.44; CA3b *p* = 0.14).

## Discussion

The formation, stabilization and retrieval of precisely timed activity of specific neuronal assemblies may represent a network-level correlate of memory. Conserved temporal relationships between discharges of selected neurons have been observed in various brain regions (Wilson and McNaughton, 1994; Pizzagalli et al., 2003; Pitzalis et al., 2013). In the present study, we made use of an *in vitro* model of hippocampal sharp wave-ripple (SPW-R) complexes. We evoked field events in mouse hippocampal slices by a sparse and distributed activation of CA3 neurons through electrical stimulation of presynaptic neurons in the upstream DG. On evoked downstream response pattern we observed lower multi-unit activity than on average spontaneous SPW-R which possibly originate from not completely resolved peri-somatic inhibition. Since different levels of multi-unit activity are also found in spontaneously occurring SPW-R and stimulation induced waveform were highly similar to spontaneous SPW-R patterns, we hoped to activate physiological neuronal assemblies in the hippocampal subfields of CA1 and CA3.

We observed that specific and repetitive activation of evoked SPW-R complexes induced a stabilization of the stimulated patterns in a region specific manner. In CA1, repetitive stimulation (0.1 Hz, 100 times) of defined patterns led to an increased stability of the evoked assembly activity. Surprisingly, we also observed a stabilization of the evoked pattern that was not repetitively stimulated. This may be explained by the large overlap between parameters of both patterns which did even increase following stimulation. Interestingly, spontaneously occurring SPW-R also appeared to be stabilized by the repetitive activation of a specific assembly, as reflected by a reduced variability of spontaneously occurring waveforms. In CA3, repetitive stimulation prevented the increased distance between evoked and spontaneous SPW-R which was observed under control conditions without repetitive stimulation.

Local field potentials are the excitatory and inhibitory postsynaptic potentials as well as action potentials of individual cells (Schomburg et al., 2012). Features of well-defined field potential waveforms can be used to investigate the underlying assembly composition *in vitro* (Reichinnek et al., 2010) and *in vivo* (Agarwal et al., 2014). Specifically, we have shown previously that the stability and plasticity of neuronal assemblies can reliably be investigated by analyzing the distinct waveforms of SPW-R complexes (Reichinnek et al., 2010; Zylla et al., 2013). While neuronal assemblies cannot be precisely defined with this method, the assembly composition of the slice as a whole can be appropriately analyzed and compared (Reichinnek et al., 2010). We strived to activate a sparse and distributed population of neurons in the CA3 region by stimulation within the supra-pyramidal blade of the dentate gyrus (DG). At this location we expect to stimulate both, DG granule cells and fibers of the perforant path which both innervate CA3 pyramidal cells. As horizontal slices only maintain about 10% of the fibers between the DG and the hippocampus proper, this method of stimulation will generate a sparse and distributed activation of postsynaptic neurons in the CA3 region (Bischofberger et al., 2006; Geiger J, personal communication).

The stabilizing effect of repetitive activation on SPW-R complexes in CA1 was dependent on NMDA receptors (suggesting a contribution of synaptic plasticity, e.g., at the Schaffer collateral synapse) and on the timing of stimulation with respect to spontaneous SPW-R. Plasticity was induced when stimuli were set at ~150 ms after a spontaneous SPW-R while stimulation during an ongoing SPW-R event was without any effect. In this case, the evoked events were clearly different from spontaneous SPW-R and showed high variability. These data suggest that stimulation during spontaneous SPWR leads to aberrant, incomplete or overlapping activation of assemblies, thereby preventing stabilization of pre-existing neuronal connections. Interestingly, neither NMDA receptor blockade nor incomplete and unspecific assembly activation altered the stabilizing effect of repetitive stimulation in the CA3 region. This finding is compatible with a role of mossy fiber synapses for changes in assemblies (Nicoll and Schmitz, 2005; Evstratova and Tóth, 2014). Thus, our results indicate different mechanisms of activity-depended assembly plasticity within the CA3 and the CA1 sub-networks.

Classical protocols for long term potentiation (LTP) at the Schaffer collateral synapse use stimulation frequencies at around 100 Hz (Larson et al., 1986; Barnes et al., 1994; Durand et al., 1996; Abraham and Huggett, 1997; Colgin et al., 2004). The same paradigm is also able to induce spontaneous SPW-R oscillations in “silent” hippocampal slices (Behrens et al., 2005). Additionally, it has been shown that stimulation frequencies below 10 Hz induce hardly any potentiation and that a 1 Hz stimulation incudes long term depression (LTD) rather than LTP (Dunwiddie and Lynch, 1978). Interestingly, memory consolidation by SPW-R complexes *in vivo* is characterized by a low frequency occurrence of SPW at 2–4 Hz (Steriade et al., 1993; Ylinen et al., 1995; Csicsvari et al., 1999; Wolansky et al., 2006; Schall et al., 2008; Mölle and Born, 2011; Sadowski et al., 2011) while each of the complexes generates several cycles of high frequency oscillations (Ylinen et al., 1995). One consequence of superimposed ripple oscillations is the highly synchronized and near coincident spiking of neurons (Wilson and McNaughton, 1994) that might provide a privileged condition for plasticity processes during SPW-R (Csicsvari et al., 1999; King et al., 1999; Girardeau et al., 2009; Sadowski et al., 2011).

Why do CA3 and CA1 behave differently? The CA3 region has been suggested to form an auto-associative network for transient storage of memories (Kesner, 2013; Rolls, 2013). Moreover, it supports pattern completion upon partial cue activation (Nakazawa et al., 2002; Kesner and Rolls, 2015). Additionally, CA3 serves as the initiator of SPW-R oscillations during sleep and might play an important role in guaranteeing the replay of stored network patterns (Buzsáki, 1989; Wilson and McNaughton, 1994). The underlying pronounced plasticity might explain the tendency of CA3 to form new assemblies following repetitive consolidation of specific sub-sets of neurons. CA1, on the other hand, forms a critical interface between the direct input of environmental information by the entorhinal cortex and the pre-processed information from the CA3 region. This region has been proposed to act as a comparator between previously stored input patterns from CA3 and the actual input coming directly from the entorhinal cortex (Vinogradova, 2001; Kumaran and Maguire, 2007). In addition, CA1 serves as a major source of outputs from hippocampal to neocortical areas and thus plays a crucial role in the transfer process of memory from the hippocampus to the neocortex (Chrobak and Buzsáki, 1994, 1996). These properties might require a higher stability of information which is reflected by both, a stable re-activation of neuronal assemblies under control condition and the tendency to stabilize pre-existing assemblies by repetitive activation.

In summary, low-frequency repetitive activation of specific fast oscillating neuronal activity patterns causes a stabilization and consolidation of the underlying neuronal assemblies. This process of network-level plasticity expresses characteristic regional differences, in line with the proposed differential functions of CA3 (pattern completion and auto-associative memory) and CA1 (novelty detection and readout of existing assemblies).

## Conflict of Interest Statement

The authors declare that the research was conducted in the absence of any commercial or financial relationships that could be construed as a potential conflict of interest.

## References

[B1] Abraham W. C. Huggett A. (1997). Induction and reversal of long-term potentiation by repeated high-frequency stimulation in rat hippocampal slices. Hippocampus 7, 137–145. 10.1002/(sici)1098-1063(1997)7:2<137::aid-hipo3>3.0.co;2-k 9136046

[B2] Acsády L. Kamondi A. Sík A. Freund T. Buzsáki G. (1998). GABAergic cells are the major postsynaptic targets of mossy fibers in the rat hippocampus. J. Neurosci. 18, 3386–3403. 9547246 10.1523/JNEUROSCI.18-09-03386.1998 PMC6792657

[B3] Agarwal G. Stevenson I. H. Berényi A. Mizuseki K. Buzsáki G. Sommer F. T. (2014). Spatially distributed local fields in the hippocampus encode rat position. Science 344, 626–630. 10.1126/science.1250444 24812401 PMC4909490

[B4] Barnes C. A. Jung M. W. McNaughton B. L. Korol D. L. Andreasson K. Worley P. F. (1994). LTP saturation and spatial learning disruption: effects of task variables and saturation levels. J. Neurosci. 14, 5793–5806. 7931545 10.1523/JNEUROSCI.14-10-05793.1994 PMC6576980

[B5] Behrens C. J. van den Boom L. P. de Hoz L. Friedman A. Heinemann U. (2005). Induction of sharp wave-ripple complexes *in vitro* and reorganization of hippocampal networks. Nat. Neurosci. 8, 1560–1567. 10.1038/nn1571 16222227

[B6] Bischofberger J. Engel D. Li L. Geiger J. R. Jonas P. (2006). Patch-clamp recording from mossy fiber terminals in hippocampal slices. Nat. Protoc. 1, 2075–2081. 10.1038/nprot.2006.312 17487197

[B7] Bliss T. V. Lomo T. (1973). Long-lasting potentiation of synaptic transmission in the dentate area of the anaesthetized rabbit following stimulation of the perforant path. J. Physiol. 232, 331–356. 10.1113/jphysiol.1973.sp010273 4727084 PMC1350458

[B8] Both M. Bähner F. Von Bohlen Und Halbach O. Draguhn A. (2008). Propagation of specific network patterns through the mouse hippocampus. Hippocampus 18, 899–908. 10.1002/hipo.20446 18493949

[B9] Buschler A. Goh J. J. Manahan-Vaughan D. (2012). Frequency dependency of NMDA receptor-dependent synaptic plasticity in the hippocampal CA1 region of freely behaving mice. Hippocampus 22, 2238–2248. 10.1002/hipo.22041 22707377

[B10] Buzsáki G. (1989). Two-stage model of memory trace formation: a role for “noisy” brain states. Neuroscience 31, 551–570. 10.1016/0306-4522(89)90423-5 2687720

[B11] Buzsáki G. Draguhn A. (2004). Neuronal oscillations in cortical networks. Science 304, 1926–1929. 10.1126/science.1099745 15218136

[B12] Carr M. F. Karlsson M. P. Frank L. M. (2012). Transient slow gamma synchrony underlies hippocampal memory replay. Neuron 75, 700–713. 10.1016/j.neuron.2012.06.014 22920260 PMC3428599

[B13] Chrobak J. J. Buzsáki G. (1994). Selective activation of deep layer (V-VI) retrohippocampal cortical neurons during hippocampal sharp waves in the behaving rat. J. Neurosci. 14, 6160–6170. 7931570 10.1523/JNEUROSCI.14-10-06160.1994 PMC6576977

[B14] Chrobak J. J. Buzsáki G. (1996). High-frequency oscillations in the output networks of the hippocampal-entorhinal axis of the freely behaving rat. J. Neurosci. 16, 3056–3066. 8622135 10.1523/JNEUROSCI.16-09-03056.1996 PMC6579047

[B15] Colgin L. L. Kubota D. Jia Y. Rex C. S. Lynch G. (2004). Long-term potentiation is impaired in rat hippocampal slices that produce spontaneous sharp waves. J. Physiol. 558, 953–961. 10.1113/jphysiol.2004.068080 15194734 PMC1665012

[B16] Csicsvari J. Hirase H. Czurkó A. Mamiya A. Buzsáki G. (1999). Oscillatory coupling of hippocampal pyramidal cells and interneurons in the behaving rat. J. Neurosci. 19, 274–287. 9870957 10.1523/JNEUROSCI.19-01-00274.1999 PMC6782375

[B17] Dunwiddie T. Lynch G. (1978). Long-term potentiation and depression of synaptic responses in the rat hippocampus: localization and frequency dependency. J. Physiol. 276, 353–367. 10.1113/jphysiol.1978.sp012239 650459 PMC1282430

[B18] Durand G. M. Kovalchuk Y. Konnerth A. (1996). Long-term potentiation and functional synapse induction in developing hippocampus. Nature 381, 71–75. 10.1038/381071a0 8609991

[B19] Evstratova A. Tóth K. (2014). Information processing and synaptic plasticity at hippocampal mossy fiber terminals. Front. Cell. Neurosci. 8:28. 10.3389/fncel.2014.00028 24550783 PMC3912358

[B20] Frank M. J. Rudy J. W. O’reilly R. C. (2003). Transitivity, flexibility, conjunctive representations and the hippocampus. II. A computational analysis. Hippocampus 13, 341–354. 10.1002/hipo.10084 12722975

[B21] Girardeau G. Benchenane K. Wiener S. I. Buzsáki G. Zugaro M. B. (2009). Selective suppression of hippocampal ripples impairs spatial memory. Nat. Neurosci. 12, 1222–1223. 10.1038/nn.2384 19749750

[B22] Goh J. J. Manahan-Vaughan D. (2013). Synaptic depression in the CA1 region of freely behaving mice is highly dependent on afferent stimulation parameters. Front. Integr. Neurosci. 7:1. 10.3389/fnint.2013.00001 23355815 PMC3555076

[B23] Kesner R. P. (2013). A process analysis of the CA3 subregion of the hippocampus. Front. Cell. Neurosci. 7:78. 10.3389/fncel.2013.00078 23750126 PMC3664330

[B24] Kesner R. P. Rolls E. T. (2015). A computational theory of hippocampal function and tests of the theory: new developments. Neurosci. Biobehav. Rev. 48c, 92–147. 10.1016/j.neubiorev.2014.11.009 25446947

[B25] King C. Henze D. A. Leinekugel X. Buzsáki G. (1999). Hebbian modification of a hippocampal population pattern in the rat. J. Physiol. 521(Pt. 1), 159–167. 10.1111/j.1469-7793.1999.00159.x 10562342 PMC2269637

[B26] Köhr G. Jensen V. Koester H. J. Mihaljevic A. L. Utvik J. K. Kvello A.. (2003). Intracellular domains of NMDA receptor subtypes are determinants for long-term potentiation induction. J. Neurosci. 23, 10791–10799. 14645471 10.1523/JNEUROSCI.23-34-10791.2003 PMC6740988

[B27] Kumaran D. Maguire E. A. (2007). Which computational mechanisms operate in the hippocampus during novelty detection? Hippocampus 17, 735–748. 10.1002/hipo.20326 17598148

[B28] Larson J. Wong D. Lynch G. (1986). Patterned stimulation at the theta frequency is optimal for the induction of hippocampal long-term potentiation. Brain Res. 368, 347–350. 10.1016/0006-8993(86)90579-2 3697730

[B29] Lee A. K. Wilson M. A. (2002). Memory of sequential experience in the hippocampus during slow wave sleep. Neuron 36, 1183–1194. 10.1016/s0896-6273(02)01096-6 12495631

[B30] Lisman J. E. Talamini L. M. Raffone A. (2005). Recall of memory sequences by interaction of the dentate and CA3: a revised model of the phase precession. Neural Netw. 18, 1191–1201. 10.1016/j.neunet.2005.08.008 16233972

[B31] Lopes-dos-Santos V. Ribeiro S. Tort A. B. (2013). Detecting cell assemblies in large neuronal populations. J. Neurosci. Methods 220, 149–166. 10.1016/j.jneumeth.2013.04.010 23639919

[B32] Maier N. Nimmrich V. Draguhn A. (2003). Cellular and network mechanisms underlying spontaneous sharp wave-ripple complexes in mouse hippocampal slices. J. Physiol. 550, 873–887. 10.1113/jphysiol.2003.044602 12807984 PMC2343079

[B33] Mölle M. Born J. (2011). Slow oscillations orchestrating fast oscillations and memory consolidation. Prog. Brain Res. 193, 93–110. 10.1016/b978-0-444-53839-0.00007-7 21854958

[B34] Nakazawa K. Quirk M. C. Chitwood R. A. Watanabe M. Yeckel M. F. Sun L. D.. (2002). Requirement for hippocampal CA3 NMDA receptors in associative memory recall. Science 297, 211–218. 10.1126/science.1071795 12040087 PMC2877140

[B35] Nicolelis M. A. Baccala L. A. Lin R. C. Chapin J. K. (1995). Sensorimotor encoding by synchronous neural ensemble activity at multiple levels of the somatosensory system. Science 268, 1353–1358. 10.1126/science.7761855 7761855

[B36] Nicoll R. A. Schmitz D. (2005). Synaptic plasticity at hippocampal mossy fibre synapses. Nat. Rev. Neurosci. 6, 863–876. 10.1038/nrn1786 16261180

[B37] O’Keefe J. Recce M. L. (1993). Phase relationship between hippocampal place units and the EEG theta rhythm. Hippocampus 3, 317–330. 10.1002/hipo.450030307 8353611

[B38] Palmer M. J. Isaac J. T. Collingridge G. L. (2004). Multiple, developmentally regulated expression mechanisms of long-term potentiation at CA1 synapses. J. Neurosci. 24, 4903–4911. 10.1523/jneurosci.0170-04.2004 15163681 PMC6729367

[B39] Pitzalis S. Bozzacchi C. Bultrini A. Fattori P. Galletti C. Di Russo F. (2013). Parallel motion signals to the medial and lateral motion areas V6 and MT+. Neuroimage 67, 89–100. 10.1016/j.neuroimage.2012.11.022 23186916

[B40] Pizzagalli D. A. Greischar L. L. Davidson R. J. (2003). Spatio-temporal dynamics of brain mechanisms in aversive classical conditioning: high-density event-related potential and brain electrical tomography analyses. Neuropsychologia 41, 184–194. 10.1016/s0028-3932(02)00148-3 12459216

[B41] Reichinnek S. Künsting T. Draguhn A. Both M. (2010). Field potential signature of distinct multicellular activity patterns in the mouse hippocampus. J. Neurosci. 30, 15441–15449. 10.1523/jneurosci.2535-10.2010 21084600 PMC6633665

[B42] Rolls E. T. (2013). A quantitative theory of the functions of the hippocampal CA3 network in memory. Front. Cell. Neurosci. 7:98. 10.3389/fncel.2013.00098 23805074 PMC3691555

[B43] Sadowski J. H. Jones M. W. Mellor J. R. (2011). Ripples make waves: binding structured activity and plasticity in hippocampal networks. Neural Plast. 2011:960389. 10.1155/2011/960389 21961073 PMC3180853

[B44] Schall K. P. Kerber J. Dickson C. T. (2008). Rhythmic constraints on hippocampal processing: state and phase-related fluctuations of synaptic excitability during theta and the slow oscillation. J. Neurophysiol. 99, 888–899. 10.1152/jn.00915.2007 18046004

[B45] Schomburg E. W. Anastassiou C. A. Buzsáki G. Koch C. (2012). The spiking component of oscillatory extracellular potentials in the rat hippocampus. J. Neurosci. 32, 11798–11811. 10.1523/jneurosci.0656-12.2012 22915121 PMC3459239

[B46] Sirota A. Buzsáki G. (2005). Interaction between neocortical and hippocampal networks via slow oscillations. Thalamus Relat. Syst. 3, 245–259. 10.1017/s1472928807000258 18185848 PMC2180396

[B47] Steriade M. McCormick D. A. Sejnowski T. J. (1993). Thalamocortical oscillations in the sleeping and aroused brain. Science 262, 679–685. 10.1126/science.8235588 8235588

[B48] Vinogradova O. S. (2001). Hippocampus as comparator: role of the two input and two output systems of the hippocampus in selection and registration of information. Hippocampus 11, 578–598. 10.1002/hipo.1073.abs 11732710

[B49] Wilson M. A. McNaughton B. L. (1994). Reactivation of hippocampal ensemble memories during sleep. Science 265, 676–679. 10.1126/science.8036517 8036517

[B50] Wolansky T. Clement E. A. Peters S. R. Palczak M. A. Dickson C. T. (2006). Hippocampal slow oscillation: a novel EEG state and its coordination with ongoing neocortical activity. J. Neurosci. 26, 6213–6229. 10.1523/jneurosci.5594-05.2006 16763029 PMC6675178

[B51] Ylinen A. Bragin A. Nádasdy Z. Jandó G. Szabó I. Sik A.. (1995). Sharp wave-associated high-frequency oscillation (200 Hz) in the intact hippocampus: network and intracellular mechanisms. J. Neurosci. 15, 30–46. 7823136 10.1523/JNEUROSCI.15-01-00030.1995 PMC6578299

[B52] Zylla M. M. Zhang X. Reichinnek S. Draguhn A. Both M. (2013). Cholinergic plasticity of oscillating neuronal assemblies in mouse hippocampal slices. PLoS One 8:e80718. 10.1371/journal.pone.0080718 24260462 PMC3832478

